# Early-warning signals for infectious diseases with a social-media compartment

**DOI:** 10.1371/journal.pone.0354091

**Published:** 2026-07-28

**Authors:** Francisca Olajide, Frithjof Lutscher, Stacey R. Smith?

**Affiliations:** 1 Department of Mathematics and Statistics, The University of Ottawa, Ottawa, Canada; 2 Department of Mathematics and Statistics, and Department of Biology, The University of Ottawa, Ottawa, Canada; 3 Department of Mathematics and Faculty of Medicine, The University of Ottawa, Ottawa, Canada; University of Dhaka, BANGLADESH

## Abstract

Early-warning signals (EWSs) are crucial tools for anticipating disease emergence and guiding public-health responses, but uncertainties in transmission and incomplete data can limit their reliability. Additionally, the performance of EWSs is rarely evaluated when disease emergence is delayed, and their use in the context of interactions between disease transmission and communication through social-media platforms has largely not been considered. We evaluate the relative performance of EWSs in predicting disease emergence under varying noise conditions and explore the use of EWSs with social-media dynamics to predict disease emergence. We develop a mechanistic model coupling infectious disease and social-media dynamics, introduce stochasticity and generate simulated time series. We detect changepoints, quantify delays relative to the bifurcation point and assess the performance of EWSs across different segments of the time series. The “reporting” infected compartment proves most reliable, and variance outperforms autocorrelation in high-noise scenarios. However, in the social-media compartment, variance and autocorrelation have weak predictive power. Our work provides a framework to advance understanding of how EWSs can be applied to forecasting disease emergence, contributing to improved disease preparedness and response.

## Introduction

Early detection of infectious diseases can provide precious time to implement effective interventions [1,2], which can mitigate the often-disastrous consequences of such diseases on various aspects of human existence [3,4]. The emergence of the SARS-CoV-2 pathogen exemplifies this impact, resulting in millions of deaths, strain on healthcare resources and far-reaching economic and social consequences, including business closures, job losses and significant disruptions to daily life [3,5]. Our work proposes and evaluates the relative efficiency of some early detection methods.

Three factors make the early detection of an emerging disease particularly difficult. (1) As with any real-world process, noise is inherent in the process of disease emergence and can hide the signal of early disease transmission when case numbers are low. (2) Data on disease emergence are often incomplete, since many infectious individuals are either unaware of their infection or do not report it. (3) Mathematical models often show a delay between the theoretical prediction and the actual observation of an emerging disease. Our work helps to quantify this delay in view of different forms of noise and reporting patterns.

The emergence of an infectious disease is often viewed as a critical transition from the absence of the disease to its presence. In mathematical models, formulated as deterministic dynamical systems, such transitions correspond to bifurcations [6,7]. Typically, the stable disease-free equilibrium becomes unstable and a stable endemic equilibrium emerges in a transcritical bifurcation, which occurs when the basic reproduction number is equal to one [4,8]. Such phenomena are widely seen in numerous models in mathematical biology [9]. The basic reproduction number (*R*_0_) is the average number of secondary infections caused by a single infectious individual in a completely susceptible population [10].

In stochastic dynamical systems, the theory of critical slowing can identify approaching critical transitions [7,11–13]. It suggests that a system gradually loses its ability to recover from perturbations as it approaches a critical transition [12,14]. Simultaneously, statistical patterns of solutions can change [15]. For example, critical slowing down leads to a decrease in the rate of change of the system, causing the current state of the system to become more correlated with its past state, thereby increasing lag-1 autocorrelation [2,7,11,16]. It also leads to increasing variance in solutions [11,13,15–17]. These and other statistical measures used to anticipate critical transitions are generally referred to as early warning signals (EWSs); they are generically applicable for identifying critical transitions [2,11,13,15–19]. Previous studies have shown that EWSs are sometimes useful in providing early indicators of disease emergence, re-emergence and elimination [2,18,20–23].

In systems where parameter values change over time, it is tempting to think that a critical transition occurs when the parameter(s) pass through the bifurcation point, but this is not necessarily true. Instead, critical transitions are typically delayed [24,25]. In the context of disease emergence, when *R*_0_ gradually increases and crosses the critical threshold of one, the disease will not emerge immediately but rather remain nearly undetectable for some time before undergoing a delayed but quick transition to the endemic state [21,26,27]. The phenomenon that system solutions stay near an unstable state before the transition occurs has been termed “tracking unstable states” [28]. Since critical transitions can be delayed and decoupled from bifurcation points, basing their predictions on bifurcations can lead to inaccurate outbreak risk assessment and poorly timed interventions.

Critical transitions manifest in time-series data as abrupt shifts, such as a sudden rise (emergence) or a sudden decline (elimination) in the number of infected individuals. They can be found using changepoint detection methods, which indicate whether and when significant shifts in the statistical properties of the data (e.g., mean, trend or variance) occur [29]. EWSs have been used to predict bifurcation points in time-series data [7,11,15]. However, when transitions are delayed, it is not clear whether EWSs can be used to predict changepoints [30]. Our work helps assess the performance of EWSs in detecting changepoints and provides a better understanding of how EWSs can be applied to epidemic forecasting.

Noise is an inherent feature of real-world processes that makes detection of critical transitions difficult. It can result from intrinsic fluctuations (demographic stochasticity) or from extrinsic forcing (environmental stochasticity) and can affect the stability of a system and the timing of critical transitions. Several studies have found that noise may increase the bifurcation delay [11,31], whereas others suggest that it may induce a transition before the bifurcation point [31,32]. Consequently, the performance of EWSs in predicting transitions can vary considerably depending on the nature and intensity of stochastic fluctuations. O’Regan and Burton [33] found that some EWSs, such as lag-1 autocorrelation and coefficient of variation, are robust to noise, whereas variance is more sensitive to the form of noise in a simple, one-dimensional system. Dutta et al. [34] found that while EWSs perform reliably in predicting saddle-node bifurcations, their performance is more sensitive to noise type when applied to other transitions.

Availability and quality of data is another factor potentially limiting the ability of EWSs to predict critical transitions. While EWSs can predict critical transitions based on disease prevalence in simple disease models [2,13,18,20,21], empirical data is often affected by inconsistent data collection, lags in case reporting, testing frequencies and underreporting [13]. EWSs generally require high-frequency data from longer time series for improved performance [35], but such high-quality data is often not available [13,32]. These limitations emphasize the need for additional data sources and proxies [32]. Previous studies have explored the use of Google searches (searches on symptoms and health-related issues) and activities (reported symptoms) from social-media platforms (Twitter, Facebook, et cetera) to enhance early warning of disease outbreaks [36]. These studies used techniques such as sentiment analysis and natural language processing. They found that Google searches and social-media data strongly correlate with disease incidence and, in some cases, precede public-health reports [36–40]. This raises the prospect of using EWSs to assess the potential of social-media dynamics in predicting disease emergence.

Here, we develop a framework that integrates delays in disease emergence, incomplete reporting and random noise, together with alternative data sources such as social media dynamics, to evaluate EWSs of infectious diseases. The objectives of our study are:

(i) Exploring the effectiveness of EWSs in predicting disease emergence in the presence of interactions between delays, noise and incomplete information.
(ii) Assessing the potential of using EWSs with partial information about infections and with social-media dynamics to predict disease emergence under different noise scenarios.

## Model derivation

### The deterministic SWIRM model

**Fig 1:**
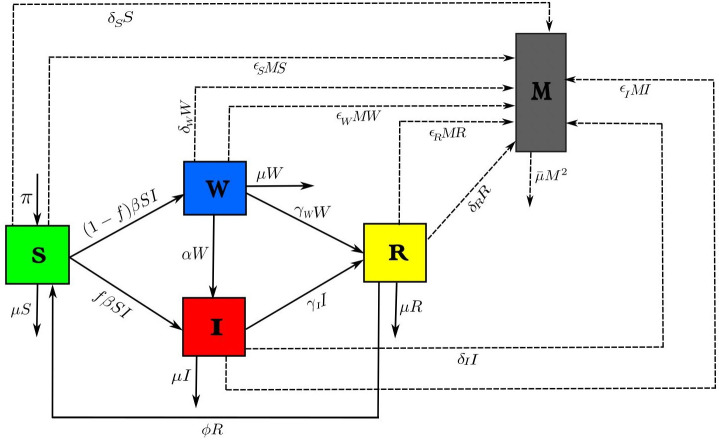
Flow diagram of the SWIRM model. Solid arrows represent transitions within the human population, while dashed arrows represent interactions with the media compartment.

We extend the classic SIR model by splitting infected individuals into two compartments and by introducing a social-media compartment (see Fig 1). We assume that not all infected individuals enter the healthcare system, and only the information from those who do is available to decision-makers. Based on this assumption, we divide infected individuals into two classes: *W* represents infected individuals who do not report to the doctor (for example because they experience only mild symptoms, although this class may include individuals with severe symptoms who do not report), and *I* represents infected individuals who do report to the doctor (for example because they have severe symptoms). Furthermore, *S* represents susceptible individuals, *R* represents recovered individuals and *M* represents social-media posts about the disease (see below).

Individuals are recruited into the susceptible compartment at a constant rate π; recovered individuals may lose their immunity at rate ϕ; susceptible individuals may become infected with transmission rate β (this rate will later depend on time). Unreported infected individuals may progress to the reported infected class at rate α; infected individuals in the *W* and *I* compartments recover at rates $\gamma_{W}$ and $\gamma_{I}$, respectively. The natural death rate μ is assumed to be the same for all the sub-populations.

Within the context of our study, the social-media compartment *M* is modelled as an observable proxy for disease dynamics rather than a mechanistic driver of behaviour or transmission. We assume that individuals in the *S*, *W*, *I* and *R* compartments generate posts at rates $\delta_{S}$, $\delta_{W}$ $\delta_{I}$ and $\delta_{R}$, and interact with existing content at rates $\epsilon_{S}$, $\epsilon_{W}$, $\epsilon_{I}$ and $\epsilon_{R}$ through engagement metrics such as likes, shares, retweets, etc. We note that this assumption is a simplification and does not fully account for the variability observed in real‑world social networks. We denote the rate at which social-media posts become less visible to users as $\bar{\mu }$. Parameter descriptions, units and values are given in Table 1.

**Table 1:** Parameters, description, units and values. $\hat{I}$ represents individuals, $\hat{T}$ represents time (days), and $\hat{P}$ represents posts. $\delta_{S},\delta_{W},\delta_{I},\delta_{R}$ are proportions of δ and $\epsilon_{S},\epsilon_{W},\epsilon_{I},\epsilon_{R}$ are proportions of ϵ. The symbol (*) means the parameters were adjusted within a range of 30−86% relative to values used in previous studies. The numerical parameter *r* was calculated such that *R*_0_(*t*) reaches the threshold value of 1 at a reasonable time point (around 14 days); π was calculated so that there are 1000 people at the disease-free equilibrium; ϵ was set to 50% of δ to reflect the tendency of users to passively consume health-related content. We chose a relatively high fraction for infected individuals who report; while this may be lower for diseases like COVID-19, it is plausible for mandatory reportable infections such as measles.

	Parameters	Description	Units	Values	Source
Demographic parameters	π	Birth rate	$\hat{I}{\hat{T}}^{-1}$	14.28	Calculated
	μ	Natural death rate	${\hat{T}}^{-1}$	0.01428	[41]^*^
Disease-related parameters	*f*	Fraction of infected individuals who report	unitless	0.8	Assumed
	β=β(t)	Transmission rate	$(\hat{I}\hat{T}{)}^{-1}$	$\beta_{0}+rt$	
	$\gamma_{W}$	Recovery rate of unreported infected individuals	${\hat{T}}^{-1}$	0.07	[42]^*^
	$\gamma_{I}$	Recovery rate of infected individuals who report	${\hat{T}}^{-1}$	0.024	Assumed
	ϕ	Rate of waning immunity	${\hat{T}}^{-1}$	0.01	[43]
	α	Transition rate from *W* to *I*	${\hat{T}}^{-1}$	0.033	[44]^*^
Media parameters	ϵ	Rate at which individuals engage or interact with already posted social media content (likes, shares, retweets, etc.)	$(\hat{I}\hat{T}{)}^{-1}$	0.25	Calculated
	δ	Rate at which individuals post about the disease and self-report symptoms	$\hat{P}(\hat{I}\hat{T}{)}^{-1}$	0.5	[44]
	$\bar{\mu }$	Fickle rate of social-media posts	$(\hat{P}\hat{T}{)}^{-1}$	0.1428	[44]^*^
Numerical parameters	$\beta_{0}$	Initial transmission rate	$(\hat{I}\hat{T}{)}^{-1}$	0.000005	[44]^*^
	*r*	Rate of change of β(t)		0.000002739	Calculated

The dynamics of the SWIRM model are then given by the system of equations


$$\begin{array}{cc} \frac{dS}{dt}= & \pi -\beta SI-\mu S+\phi R,\\ \frac{dW}{dt}= & (1-f)\beta SI-\gamma_{W}W-\alpha W-\mu W,\\ \frac{dI}{dt}= & f\beta SI+\alpha W-\gamma_{I}I-\mu I,\\ \frac{dR}{dt}= & \gamma_{W}W+\gamma_{I}I-\mu R-\phi R,\\ \frac{dM}{dt}= & \delta_{S}S+\delta_{W}W+\delta_{I}I+\delta_{R}R+\epsilon_{S}MS+\epsilon_{W}MW+\epsilon_{I}MI+\epsilon_{R}MR-\bar{\mu }M^{2}. \end{array}$$ (1)

The disease-free equilibrium of (1) is given by


$$\begin{array}{c} (\bar{S},\bar{W},\bar{I},\bar{R},\bar{M})=\left(\frac{\pi }{\mu },0,0,0,\frac{\epsilon_{S}\frac{\pi }{\mu }+\sqrt{{\left(\epsilon_{S}\frac{\pi }{\mu }\right)}^{2}+\frac{4\bar{\mu }\delta_{S}\pi }{\mu }}}{2\bar{\mu }}\right). \end{array}$$

We observe that even though $\bar{W}=\bar{I}=0$, we have $\bar{M}\neq 0$, which means that there are social-media posts about the disease even when there is no disease. We consider this relevant in the context where the disease is already in a different population than the one we are considering, such as in a different city or country.

We linearize the system around the disease-free equilibrium to analyze its local stability and determine the basic reproduction number; for details, see the Appendix. The basic reproduction number for our model is given by


$$\begin{array}{c} R_{0}=\frac{\alpha (1-f)\beta \frac{\pi }{\mu }+f\beta \frac{\pi }{\mu }(\gamma_{W}+\alpha +\mu )}{\left(\gamma_{W}+\alpha +\mu )(\gamma_{I}+\mu \right)}. \end{array}$$ (2)

Although a social-media compartment is included in the SWIRM model, it does not appear in the expression for the basic reproduction number, because it is a higher-order term and hence not present in the linearisation used to derive *R*_0_. It thus serves as an observable variable.

At *R*_0_ = 1, system (1) undergoes a transcritical bifurcation, and the critical transmission rate is


$$\begin{array}{c} \beta =\frac{\left(\gamma_{W}+\alpha +\mu )(\gamma_{I}+\mu \right)}{\frac{\pi }{\mu }(\alpha (1-f)+f(\gamma_{W}+\alpha +\mu ))}. \end{array}$$ (3)

In real systems, parameters such as transmission rate change slowly over time [24,25,30]. To allow the transmission rate to change over time, we write β as β(t). Substituting this function into Eq (2), we define a time-dependent reproduction number *R*_0_(*t*). We then obtain a bifurcation point in time by calculating *t* such that *R*_0_(*t*)=1.

While the function β(t) may take different functional forms, we adopt a linearly increasing function ($\beta (t)=\beta_{0}+rt$) as a simple and interpretable forcing scenario that allows the relationship between threshold crossing and the observed transition dynamics to be examined precisely. Here, $\beta_{0}>0$ is the initial transmission rate, and *r* (0<r≪1) is the rate of change in $\beta_{0}$. With this choice and the parameter values in Table 1, the bifurcation point is *t* = 14.5 days.

### Models with noise

We use model (1) as the deterministic skeleton to which we add three different forms of stochasticity: additive white noise, multiplicative white noise, and demographic stochasticity. From each of these models, we then generate time-series data that we analyze for early warning signals.

Additive white noise represents random fluctuations that do not mechanistically influence the deterministic components [45]; they are independent of population size. To include additive white noise, we add the increment of a Wiener process (denoted by W(t)) with a certain intensity to each of the equations in (1). For simplicity, we choose the same intensity (denoted by σ) for all equations.

Unlike additive white noise, which is independent of population size, multiplicative white noise models environmental fluctuations whose magnitude scales with population size [16,46]. As above, we add the increment of a Wiener process, but this time, we choose the intensity to be proportional to the population size in the respective compartments. For simplicity, we assume that the proportionality constant is the same for all compartments (again denoted by σ).

Demographic stochasticity refers to the random variation in population size that results from births, deaths, immigration and other population processes. We follow the approach by Greenwood and Gordillo [47,48]. Unlike the other forms of stochasticity, the intensity of demographic stochasticity is determined by the rates of the deterministic model (1).

We note that while there is a “free” parameter that controls the intensity of additive and multiplicative noise, no such parameter exists in the case of demographic stochasticity. Hence, we can (and will) explore the effect of noise intensity on early-warning signals in the first two cases but not in the third.

From each of the three stochastic models described here, we generate 300 time series, using the Euler–Maruyama method [49]. This method is a simple variant of the classical forward Euler method in which the increment of a Wiener process is drawn from a standard normal distribution and multiplied by the size of the timestep of the method. We use a small timestep (*dt* = 0.005) to reduce discretization error and ensure numerical stability. To maintain biological feasibility, we correct any non-physical values arising from the discretization by reverting to the previous state. For completeness, we present all the equations for all three forms of stochasticity explicitly in the Appendix.

### Methods

Next, we describe the methods used to generate and analyze time-series data for changepoints and early-warning signals, as well as the visualization techniques employed to present the results.

### Changepoint analysis

We analyze each of the time series using changepoint analysis. Specifically, we search for changepoints in the time-series data by using the mean changepoint detection (cpt.mean) function of the “changepoint” package in R [29,50]. Using the At Most One Changepoint (AMOC) method, the null hypothesis (*H*_0_) is that there are no changepoints in the data, while the alternative hypothesis (*H*_1_) is that there is exactly one changepoint in the data. The single changepoint is estimated using the “Asymptotic” penalty with pen.value = 0.05, which corresponds to a theoretical type I error of 0.05. We tested some of our results with different values of parameter pen.value and found no difference in outcome.

The cpt.mean function relies on assumptions of independence of observations within each segment, normally distributed errors and abrupt, rather than gradual, changes in the mean. In the simulated time-series data, these assumptions are generally satisfied, with the exception of independence within segments, which is violated due to autocorrelation. Based on the objectives of our study, identifying changepoints using cpt.mean represents one component of the overall pipeline, rather than the primary focus. Alternative packages, such as “envcpt” in R, account for autocorrelation and may provide more reliable changepoint detection in time-series data where the assumption of independence is violated. However, changepoint detection with “envcpt” is sensitive to the choice of minimum segment length, which can affect both the number and the location of detected changepoints. Some limitations of the changepoint package are that it may fail to detect changepoints when the underlying process changes gradually, and high noise levels can lead to spurious detections or obscure true changepoints.

**Fig 2:**
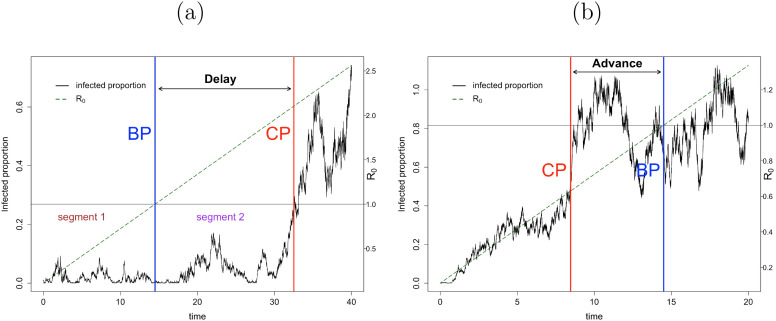
(a) Illustration of a “delay” based on a realization of the infected population in the model with demographic stochasticity. The transmission parameter is slowly forced through the bifurcation point *R*_0_ = 1 (the blue line). The red line representing the changepoint is computed using the cpt.mean function in R. The delay occurs between the system crossing the bifurcation point (i.e., *R*_0_ = 1) and the subsequent spike in infections. Since the transmission rate varies continuously in time, crossing the threshold does not immediately produce a sharp increase in infections. (b) Illustration of an “advance” based on a realization of the infected population.

We check whether the changepoint (CP) in the time series occurs before or after the bifurcation point (BP). If the CP occurs after the BP, we define the scenario as a delay (Fig 2(a)); conversely if the CP precedes the BP, we define the scenario as an advance (Fig 2(b)).

We note that the BP is obtained analytically; therefore, any sensitivity to timestep size is expected to arise through the estimation of changepoints and the corresponding delay values. To clarify this, we carry out additional simulations for the lowest noise intensity case under additive white noise using timesteps *dt* = 0.0025 and *dt* = 0.01 in the Appendix.

### Early-warning signals

We use the “earlywarnings” package in R to calculate EWSs for a given time series. More specifically, we calculate the autocorrelation and variance of a time series and compute the Kendall-τ correlation coefficient (ranging from −1 to +1) to detect trends in these quantities. Positive and negative Kendall-τ values represent an increasing and decreasing trend of each signal, respectively, and increasing trends of autocorrelation and variance are indicators of an upcoming transition [7,11,12,31].

Since we want to compare the performance of EWSs regarding the changepoint (CP) and the bifurcation point (BP), we select two segments from each time series that show the expected delay (i.e., CP succeeds BP; see previous section). The two segments are of equal length: the length of the time series from the beginning to the BP (2900 data points). In line with standard practice [11,51], we apply Gaussian detrending to each segment to eliminate long-term trends that may obscure short-term variations. Then we use a rolling window of 50% of the detrended time-series data. Since the transition occurs at the CP and not at the BP, the ideal EWS is negative for the time series leading up to the BP and positive for the time series leading up to the CP. If the Kendall-τ value is positive and there is no transition (BP), we say it is a false positive. If there is a transition (CP) and the Kendall-τ value is positive, we say it is a true positive. Similarly, a negative Kendall-τ value despite a transition (CP) is a false negative while a negative Kendall-τ value with no transition (BP) is a true negative.

We use raincloud plots to visualize the distribution of Kendall-τ values for autocorrelation and variance. Raincloud plots provide a robust method for data visualization, incorporating a density plot (“the cloud”), raw data observations (“the rain”) and a box and whisker plot [52].

To compare the performance of autocorrelation and variance in predicting CPs, we use receiver-operating characteristic (ROC) curves. An ROC curve plots the true positive rate against the false positive rate at various threshold settings. We use the area under the curve (AUC) to measure the performance of autocorrelation and variance in predicting the changepoint. A higher AUC indicates a better EWS. An AUC of 1 means that the EWS perfectly identifies the positives and negatives, while an AUC of 0.5 indicates that the EWS performs no better than random guessing.

## Results

We begin by reporting whether there is a delay or an advance in each compartment; i.e., whether the changepoint (CP) is after or before the bifurcation point (BP). We also compare the changepoints between the different compartments; that is, whether the transitions in one compartment occur before or after the transitions in the other compartment. These results help us identify compartments that can be used to detect transitions early or predict other compartments.

Then we present our results regarding which EWSs (autocorrelation or variance) from which compartment (*I*, *W* or *M*) correctly predict the changepoint or incorrectly predict the bifurcation point. We reiterate that social-media posts do not influence transmission in our model, so all effects of *M* are observed through the EWSs. We present one example of results on these EWSs with raincloud plots and summarize the remaining results from each compartment for the different forms and intensities of noise using ROC curves and AUC values. These results will help us determine which EWSs have the best performance (high AUC values) and which compartment gives the strongest signal.

### Changepoints — Additive white noise

In the model with additive white noise (see eq. (5)), almost all time series show the expected delays for all values of σ; i.e., the detected CPs in all time series occur significantly after the parameter passed the BP. The only exception of an advance occurs in a time series of the *M* compartment when σ=3. Different compartments experience different delays.

**Fig 3:**
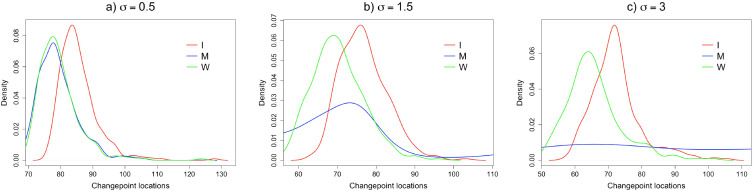
Density of changepoint locations detected for 300 time series generated from the model with additive white noise when (a) σ=0.5, (b) σ=1.5, (c) σ=3. Other parameters are as in Table 1.

The detected CPs for *W* precede those for *I* across the different noise intensities. This may indicate that the *W* compartment responds more rapidly to changes in system dynamics before these changes become pronounced in the *I* compartment. The occurrence of the detected CPs for *M*, relative to those for *I*, depends on the intensity of noise. As noise intensity increases, more and more CPs for *M* occur later than those for *I*, indicating a higher sensitivity of the *M* compartment to noise (see Fig 3). For example, with σ=0.5, the CPs for *M* precede the CPs for *I* in all time series. When σ=1.5, the CPs for *M* occur before the CPs for *I* in 78% of the time series. With σ=3, only 42% of the time series show that the CPs for *M* occur before the CPs for *I*. Time series where the CPs for *M* precede those for *I* suggest a potential connection between social-media dynamics and infection trends.

The proximity of the CPs for *M* and *W* suggests a potential association between active social-media posting and the presence of mild infectious cases, with this pattern being more apparent under lower stochastic fluctuations. As σ increases, the CPs for *M* occur substantially later than those for *W*, suggesting that the association between the two variables becomes weaker at higher noise levels.

### Changepoints — Multiplicative noise

In the model with multiplicative noise, time series for *I* and *W* show the expected delay for all values of σ. Time series for *M* show mostly delays, with advances occurring in no more than 2% of the time series across the different noise intensities.

**Fig 4:**
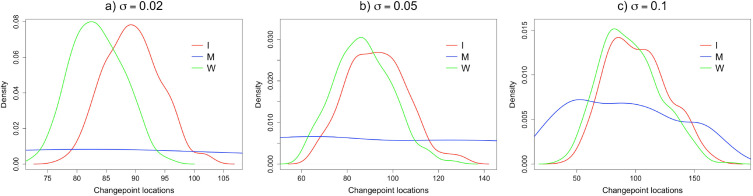
Density of changepoint locations detected for 300 time series generated from the model with multiplicative noise when (a) σ=0.02, (b) σ=0.05, (c) σ=0.1.

The CPs for *W* generally precede those for *I* (see Fig 4). However, we observe a few instances where this is not the case, particularly for higher noise intensity (here σ=0.1). As noise intensity increases, there are more instances where the CPs for *M* occur before the CPs for *I* — a result that contrasts with the observation from the model with additive white noise. This may be due to the state-dependent nature of the multiplicative noise.

Unlike the model with additive white noise, social-media dynamics show no association with mild infectious cases (Fig 4). Overall, these results suggest that the timing of changepoints across variables depends on the form and intensity of noise in the system.

### Changepoints — Demographic Stochasticity

In the model with demographic stochasticity, time series show more advances than delays. Time series for *I* show 30% delays and 70% advances; time series for *M* show 28% delays and 72% advances; and time series for *W* show 32% delays and 68% advances. These advances may be due to the fact that demographic noise can lead to the amplification of early infections.

**Fig 5:**
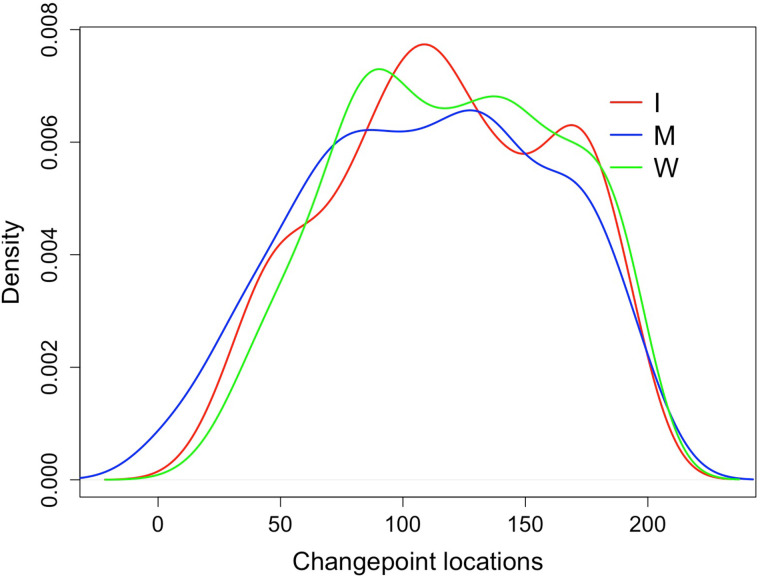
Density of changepoint locations detected for 300 time series generated from the model with demographic stochasticity.

The occurrence of the CPs for the compartments varies. In some cases, the detected CPs for *W* and *M* occur close to each other while those for *I* occur much later, whereas in other instances, the CPs for *I* align with those for *W* or *M* (see Fig 5). This is not unexpected, because the time series for the different compartments show advances and delays at different times.

### EWSs — Raincloud plots

**Fig 6:**
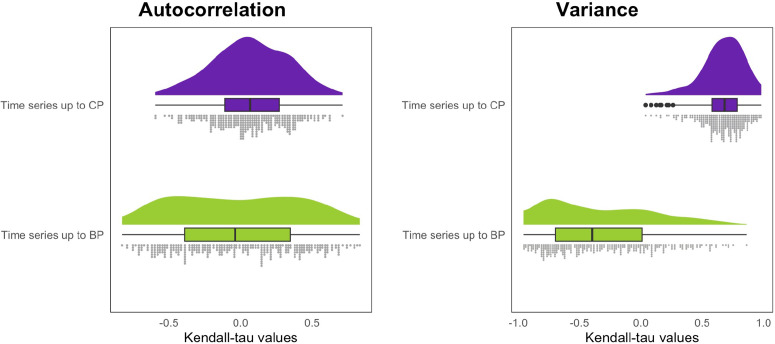
Raincloud plots showing Kendall-τ values for autocorrelation and variance, computed on time series of *I* generated from the model with multiplicative noise (σ=0.02). The black dots in the right figure represent outliers (data points beyond the whiskers of the boxplot: Q1−1.5×IQR,Q3+1.5×IQR).

In the lowest noise-intensity case of the multiplicative noise, autocorrelation (lag-1) shows 48% and 62% positives for time series of *I* leading up to the bifurcation point (BP) and changepoint (CP), respectively. Hence, autocorrelation correctly predicts CP in 62% of cases (true positives) but fails to predict in 38% of cases (false positives) (Fig 6, left). Variance computed for time series leading up to the BP shows 26% positives for *I*. Since no transition is occurring (CP occurs after BP), these are false positives. Conversely, variance computed for time series leading up to the CP shows 100% positives, correctly predicting true transitions in the time series (Fig 6, right).

Variance computed on time series of *W* shows similar result as time series of *I* in detecting true positives. However, this also results in a high number of false positives. With autocorrelation, not all true positives are identified, but there are fewer false positives compared to time series of *I*. See Table 2 for all numerical values. Autocorrelation and variance computed on time series of *M* show similar performance: not all true positives are identified, and false positives occur in roughly half of the time series (see Table 2). The remaining noise-intensity cases of the multiplicative noise show qualitatively similar results.

**Table 2:** Positive Kendall-τ values (%) for autocorrelation and variance computed prior to BP and prior to CP for the different stochastic models. AWN stands for additive white noise, MN stands for multiplicative noise and DS stands for demographic stochasticity. The best outcome in each circumstance is bolded.

Noise type	Variable	Noise intensity	Autocorrelation		Variance	
			BP	CP	BP	CP
AWN	*I*	low	45	59	42	**100**
		medium	49	**78**	51	93
		high	58	71	58	81
	*M*	low	53	**65**	56	**68**
		medium	55	45	56	46
		high	55	49	55	47
	*W*	low	53	**72**	55	**85**
		medium	58	51	61	55
		high	55	54	58	55
MN	*I*	low	48	**62**	26	**100**
		medium	51	51	33	99
		high	51	49	28	95
	*M*	low	**50**	45	49	45
		medium	46	47	47	**50**
		high	**50**	44	48	48
	*W*	low	13	**55**	96	**100**
		medium	28	49	**100**	98
		high	37	41	99	96
DS	*I*		46	**55**	48	69
	*M*		49	39	53	42
	*W*		45	**55**	43	**73**

Since many of the time series generated from the model with demographic stochasticity show advances, we could only use fewer time series for our summary; namely, those time series that showed delays.

### EWSs — Receiver-Operating Characteristic (ROC) curves

**Fig 7:**
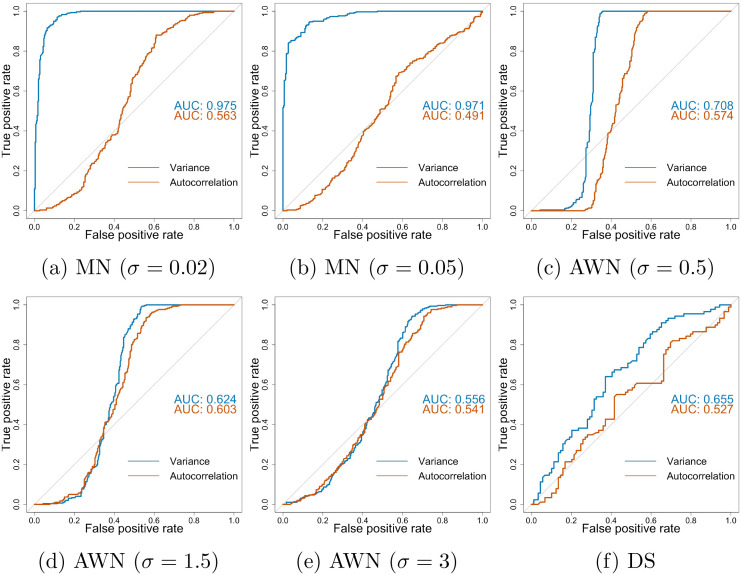
ROC curves comparing the performance of variance (blue) and autocorrelation (orange) in predicting the changepoints for *I.* The grey diagonal line represents a random classifier (AUC = 0.5). MN stands for multiplicative noise, AWN stands for additive white noise and DS stands for demographic stochasticity.

The effectiveness of the EWSs in correctly identifying transitions in time series of *I* varies depending on the form of noise and its intensity. Variance has better predictive performance than autocorrelation, with almost perfect area under the curve (AUC) values for the model with multiplicative noise (Figs 7(a)–(b)). In the lowest noise-intensity case of the model with additive white noise, variance demonstrates a moderate predictive performance (AUC ≈0.71), outperforming autocorrelation, whose performance is closer to random guessing (AUC ≈0.57); see Fig 7(c). Variance and autocorrelation show similar performance for the remaining noise-intensity cases of the model with additive white noise (Figs 7(d)–(e)). For the models with additive or multiplicative white noise, the AUC for variance decreases as noise intensity increases (Figs 7(a)–(e)). Variance outperforms autocorrelation in the model with demographic stochasticity (Fig 7(f)), but note that these results are obtained from fewer time series than in the other two cases because the model with demographic stochasticity produced more time series with advances.

**Fig 8:**
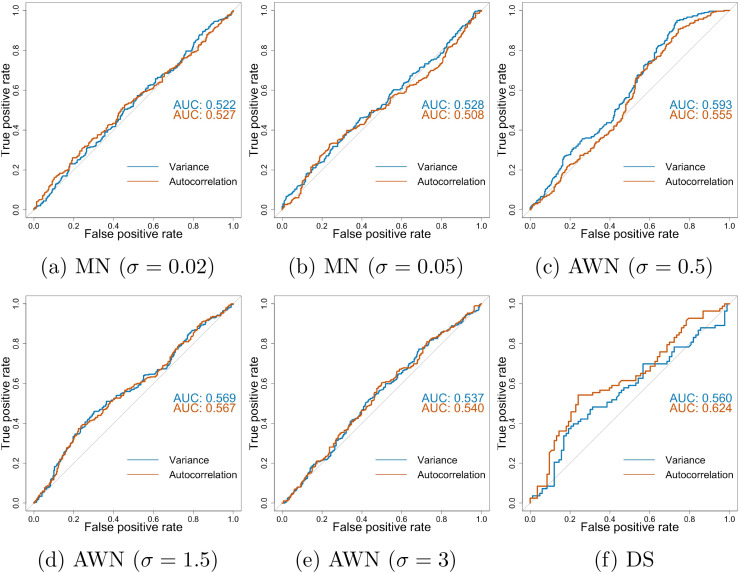
ROC curves comparing the performance of variance (blue) and autocorrelation (orange) in predicting the changepoint for *M.*

The high sensitivity of the social-media compartment to noise diminishes the detectability of the EWSs. For the models with additive or multiplicative white noise, variance and autocorrelation show similar performance, with AUC slightly above 0.5. Regardless of the noise intensity, variance and autocorrelation perform no better than random guessing in correctly predicting the transitions for the media compartment (Figs 8(a)–(e)). In the model with demographic stochasticity, autocorrelation demonstrates a slightly better predictive performance (AUC ≈0.62) compared to variance (AUC ≈0.56), although both reflect limited predictive ability (see Fig 8(f)).

**Fig 9:**
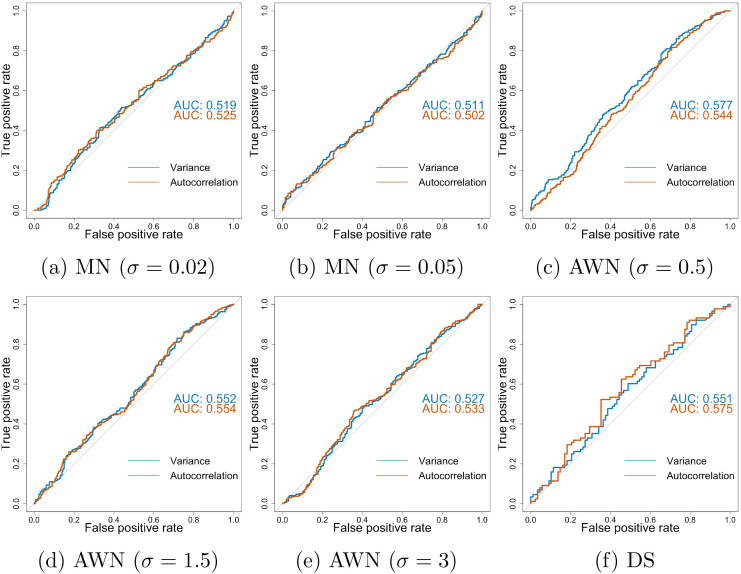
ROC curves comparing the performance of variance (blue) and autocorrelation (orange) in predicting the changepoint for *M.*

To further evaluate the sensitivity of our conclusions regarding the performance of the early warning signals (EWSs) on social‑media time‑series data, we repeated the simulations with the post‑generation rates $\delta_{S},\delta_{W},$ and $\delta_{R}$ set to zero. This restriction limited postings to only the “reporting” infected population. The resulting receiver-operating characteristic (ROC) curves are shown in Fig 9, providing a comparative assessment of EWSs’ performance under modified conditions. A comparable outcome was observed in the performance of EWSs when posts were generated exclusively by the “reporting” infected population *I*.

**Fig 10:**
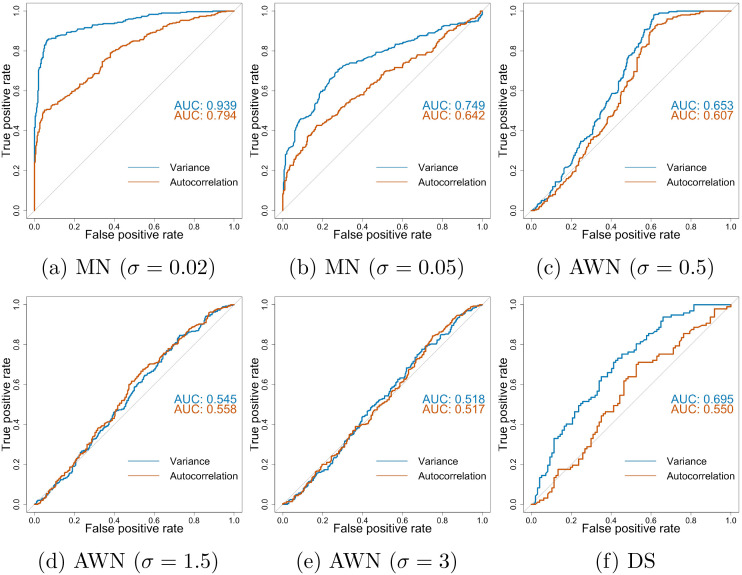
ROC curves comparing the performance of variance (blue) and autocorrelation (orange) in predicting the changepoint for *W.*

The performance of the EWSs in identifying transitions in the *W* compartment varies depending on the form of noise and noise intensity. In the lowest noise-intensity scenario of the model with multiplicative noise, variance and autocorrelation demonstrate better predictive performance, and variance outperformed autocorrelation with AUCs of 0.94 and 0.79, respectively (Fig 10(a)). As noise intensity increases, the AUC values for variance and autocorrelation decrease, indicating a decline in their predictive performance (Fig 10(a)–(b)). For higher noise intensities of the model with multiplicative noise, variance correctly identifies the true positives but also generates many false positives for the *W* compartment. Variance and autocorrelation demonstrate similar performance for the model with additive white noise, and their AUC values decrease as noise intensity increases (Figs 10(c)–(e)). In the model with demographic stochasticity (Fig 10(f)), variance demonstrates a better predictive performance (AUC ≈0.7) than autocorrelation (AUC ≈0.55).

## Discussion

### Summary

Anticipating disease emergence using early warning signals (EWSs) is crucial for public-health preparedness. However, several factors can substantially affect the precision and reliability of EWSs. Our goal was to assess different components of disease dynamics while including a social-media compartment as a proxy, to determine whether they can be used to forecast disease emergence under various scenarios using EWSs and identify which were the most reliable. We used a minimal mechanistic model to study the general pattern of disease emergence, rather than focusing on a specific disease. We implemented different forms and intensities of noise to understand the effect of noise on EWSs. We also determined which compartments gave the least delays (i.e., the earliest transitions). Finally, we analyzed the potential of social-media dynamics in predicting disease emergence. We used statistical techniques to assess and compare the performance of EWSs in these different scenarios.

### Advance and delays

Many of our stochastic simulations showed the expected delay. This result is in agreement with the findings of Dibble et al. [26] and O’Regan and Drake [21], who reported a time lag between the system crossing the critical threshold and the onset of an outbreak. However, we found — particularly for the model with demographic stochasticity — that transitions can be induced before critical thresholds are reached, resulting in advances rather than delays. In the context of delays, we found that the “non-reporting” infected population (*W*) modelled with additive white noise or multiplicative noise exhibited earlier transitions than the “reporting” infected population (*I*). Since time-series data for the former is not available, we checked whether additional information can be obtained from the social-media compartment. The hypothesis that transitions in the social-media compartment may reflect transitions in the non-reporting infected population was only supported by the model with additive white noise (lowest noise-intensity case). Hence, the possibility that transitions in the infected populations can be predicted by transitions in the social-media compartment is greatly compromised by noise.

### Performance of EWSs

The performances of variance and autocorrelation (lag-1) vary based on the form and intensity of noise. For the reporting infected population, variance had almost perfect area under the curve (AUC) with multiplicative noise, whereas the performance was moderate with additive white noise. However, its accuracy in distinguishing between true and false positives, as measured by the AUC, reduces as noise intensity increases. A similar pattern in the EWSs was observed for the non-reporting infected population, but to a lesser degree. Our observation that the form of noise affects the performance of EWSs is in partial agreement with the findings of O’Regan and Burton [33]. Using a one-dimensional system, they found that trend in variance as indicator of disease elimination is sensitive to the underlying noise structure, but autocorrelation is robust to the different forms of noise. Chakraborty et al. [53] also found that autocorrelation outperformed variance in predicting disease emergence for different noise-induced models except for the SIR model with multiplicative noise. While these studies reported autocorrelation as a more reliable EWS than variance, other studies [2,18,54] have identified variance as the better EWS. In our case, autocorrelation performed poorly in indicating disease emergence, and variance was a more reliable EWS. Further work is necessary to evaluate when these EWSs perform well based on model complexity and dimensionality.

### EWSs with social media

Although many studies have demonstrated the potential of social-media data and Google searches to predict infection trends using techniques in deep learning and natural language processing [36–38,55], the application of EWSs with social-media data in this context has not been explored. Our results showed that both variance and autocorrelation performed no better than random chance for predictions of disease emergence from the social-media compartment, regardless of the form and intensity of noise. This poor performance suggests that EWSs are more sensitive to the underlying social-media dynamics than variations in noise structure. Using a coupled behaviour-disease model with additive white noise, Pananos et al. [56] demonstrated that EWSs were effective in detecting a decline in vaccine uptake as a proxy for re-emergence of measles outbreak. To enhance the performance of EWSs in this regard, it may be necessary to account for additional critical factors, such as behavioural patterns, within the broader context of social-media activities.

### Implications of delay

Delays may underestimate the risk of a disease outbreak over an extended period, thereby limiting the window for early and effective interventions. Using systems that exhibited delays, O’Regan and Drake [21] found that EWSs failed to predict disease emergence based on pre-bifurcation time-series data. This suggests the possibility of missed detections when transitions are delayed, underscoring the importance of continuous and adaptive monitoring of disease progression. While EWSs for disease models have typically been calculated close to the bifurcation point, our study shows that they are sometimes useful in anticipating transitions that happen later.

### Potential validation strategy

Our study is based on simulated time-series data. However, to assess the performance of EWSs using past outbreaks, a feasible approach would be to consider high-resolution incidence data from diseases with known changes in transmission or interventions. The time-varying reproduction number $R_{t}$ can be reconstructed using methods such as EpiEstim with an appropriate serial-interval distribution [53,57,58]. The same data can be analyzed for changepoints, and EWSs can be computed on segments of the time series leading up to both the $R_{t}$ and the changepoint. Since we modelled the social-media compartment as a data source, EWSs could also be applied directly to tweets about the disease or its symptoms, following the same framework. (See, for example, Pananos et al. [56]).

## Limitations

Despite the insights provided by our study, some limitations should be acknowledged. First, our model assumes that the entire population are social-media users and that there is uniformity in user behaviour. This does not fully reflect the heterogeneity observed in real-world social networks [37]. Incorporating structure into social-media dynamics and exploring parameter choices based on empirical data could provide a better representation for future studies on social-epidemic models and EWSs. Second, the data for our study was generated with specific parameters. These parameters were selected based on the objective of our study, which was to determine whether we can forecast disease emergence under specific yet representative conditions using EWSs. This approach allowed us to focus on the performance of EWSs, rather than on possible variations due to extensive parameter choices. While additional sensitivity analyses could be performed, they are beyond the scope of the present study and would be more appropriately undertaken in a disease-specific setting, which we will investigate in future work. Third, we used a specific length of time-series data to evaluate the performance of EWSs. Our choice was based on the exact length of time series leading up to the bifurcation point. EWSs generally require high-frequency data from longer time series for improved performance [2,34,35]. A practical limitation is that sufficiently long time-series data is often unavailable when a new disease is emerging. It would be helpful to examine how the performance of EWSs compares with shorter time-series data. Finally, we used identical noise intensity across compartments for additive and multiplicative noise, which were chosen for comparison purposes, but which may be restrictive.

### Recommendations

Our study highlights that variance as an EWS can be used to predict disease emergence from reported infected cases. We recommend that public-health authorities prioritize improving the timeliness and quality of reporting while strengthening monitoring efforts. This will increase the effectiveness of early disease prediction, enabling quicker and more targeted measures to mitigate disease outbreaks. One of the results of our study is that noise diminishes the performance of EWSs. Future studies should evaluate the performance of other existing EWSs using complex epidemiological models. Additionally, more robust EWSs are needed for real-world applications, where noise characteristics are often unknown or heterogeneous. Our findings in using EWSs to predict disease emergence from social media suggest that the reliability of this approach may be highly context-dependent. Future work should evaluate this approach across different diseases and clarify the effect of the underlying dynamics on how EWSs reflect disease trends from social-media activities. These aspects will further aid our understanding on how EWSs can be used with social-media dynamics as a complementary tool for early disease detection within public-health settings.

## Supporting information

S1 Appendix Appendix. (PDF)
