# Supplementary material for: Early-warning signals for infectious diseases with a social-media compartment
Source: PLoS One. 2026 Jul 28;21(7):e0354091. doi: 10.1371/journal.pone.0354091 (PMC13411938; doi:10.1371/journal.pone.0354091)
Supplement: S1 Appendix — (PDF) [file pone.0354091.s001.pdf]

Appendix

Local Stability Analysis and R_0

To assess local stability of our deterministic model (1), we linearize the system around the disease-free equilibrium (DFE). The Jacobian at the DFE is

$$J|_{DFE} = \begin{bmatrix} -\mu & 0 & -\beta\frac{\pi}{\mu} & \phi & 0 \\ 0 & -(\gamma_W+\alpha+\mu) & (1-f)\beta\frac{\pi}{\mu} & 0 & 0 \\ 0 & \alpha & f\beta\frac{\pi}{\mu}-\gamma_I-\mu & 0 & 0 \\ 0 & \gamma_W & \gamma_I & -(\mu+\phi) & 0 \\ \delta_S+\epsilon_S\bar{M} & \delta_W+\epsilon_W\bar{M} & \delta_I+\epsilon_I\bar{M} & \delta_R+\epsilon_R\bar{M} & \epsilon_S\frac{\pi}{\mu}-2\bar{\mu}\bar{M} \end{bmatrix}.$$

The characteristic equation is given by

$$\left(\epsilon_S\frac{\pi}{\mu}-2\bar{\mu}\bar{M}-\lambda\right)(-\mu-\lambda)(-(\mu+\phi)-\lambda)\begin{vmatrix} -(\gamma_W+\alpha+\mu)-\lambda & (1-f)\beta\frac{\pi}{\mu} \\ \alpha & f\beta\frac{\pi}{\mu}-\gamma_I-\mu-\lambda \end{vmatrix} = 0.$$

The first three eigenvalues are negative:

$$\begin{aligned} \lambda_1 &= -\sqrt{\left(\epsilon_S\frac{\pi}{\mu}\right)^2 + 4\bar{\mu}\delta_S\frac{\pi}{\mu}}, \\ \lambda_2 &= -\mu, \\ \lambda_3 &= -(\mu+\phi). \end{aligned}$$

The remaining two eigenvalues, λ_4 and λ_5, are derived as follows:

$$\det\begin{bmatrix} -(\gamma_W+\alpha+\mu)-\lambda & (1-f)\beta\frac{\pi}{\mu} \\ \alpha & f\beta\frac{\pi}{\mu}-\gamma_I-\mu-\lambda \end{bmatrix} = 0.$$

$$(-(\gamma_W+\alpha+\mu)-\lambda)\left(f\beta\frac{\pi}{\mu}-\gamma_I-\mu-\lambda\right)-\alpha\left((1-f)\beta\frac{\pi}{\mu}\right)=0$$

Solving the above equation yields:

$$\begin{aligned} \lambda = \frac{1}{2}\Bigg[& -\gamma_W-\alpha-2\mu-\gamma_I+f\beta(t)\frac{\pi}{\mu} \\ & \pm\sqrt{\left(\gamma_W+\alpha+2\mu+\gamma_I-f\beta(t)\frac{\pi}{\mu}\right)^2+4\left((\gamma_W+\alpha+\mu)\left(f\beta(t)\frac{\pi}{\mu}-\gamma_I-\mu\right)+\alpha(1-f)\beta(t)\frac{\pi}{\mu}\right)}\Bigg], \end{aligned}$$

which then implies that λ_4 and λ_5 are negative iff

$$\frac{\alpha(1-f)\beta\frac{\pi}{\mu}+f\beta\frac{\pi}{\mu}(\gamma_W+\alpha+\mu)}{(\gamma_W+\alpha+\mu)(\gamma_I+\mu)} < 1. \tag{4}$$

The DFE is locally asymptotically stable if (4) holds.

The basic reproduction number R_0: We determine R_0 by using the next-generation method. We consider the infected classes W and I and the equations of $\dot{W}$ and $\dot{I}$ are expressed as follows:

$$\begin{aligned}\dot{W} &= \mathbb{F}_1 - \mathbb{V}_1,\\ \dot{I} &= \mathbb{F}_2 - \mathbb{V}_2,\end{aligned}$$

where $\mathbb{F}_1$ and $\mathbb{F}_2$ represent new infections and $\mathbb{V}_1$ and $\mathbb{V}_2$ denote transitions between the compartments. Then the matrix of new infections, F, and the matrix of transition between the compartments, V, are written as follows:

$$F = \begin{pmatrix} \dfrac{\partial \mathbb{F}_1}{\partial W} & \dfrac{\partial \mathbb{F}_1}{\partial I} \\ \dfrac{\partial \mathbb{F}_2}{\partial W} & \dfrac{\partial \mathbb{F}_2}{\partial I} \end{pmatrix}, \quad V = \begin{pmatrix} \dfrac{\partial \mathbb{V}_1}{\partial W} & \dfrac{\partial \mathbb{V}_1}{\partial I} \\ \dfrac{\partial \mathbb{V}_2}{\partial W} & \dfrac{\partial \mathbb{V}_2}{\partial I} \end{pmatrix}.$$

At the DFE,

$$F = \begin{pmatrix} 0 & (1-f)\beta\dfrac{\pi}{\mu} \\ 0 & f\beta\dfrac{\pi}{\mu} \end{pmatrix}$$

and

$$V = \begin{pmatrix} \gamma_W + \alpha + \mu & 0 \\ -\alpha & \gamma_I + \mu \end{pmatrix}.$$

Therefore,

$$FV^{-1} = \begin{pmatrix} \dfrac{\alpha(1-f)\beta\dfrac{\pi}{\mu}}{(\gamma_W + \alpha + \mu)(\gamma_I + \mu)} & \dfrac{(1-f)\beta\dfrac{\pi}{\mu}}{\gamma_I + \mu} \\ \dfrac{\alpha f\beta\dfrac{\pi}{\mu}}{(\gamma_W + \alpha + \mu)(\gamma_I + \mu)} & \dfrac{f\beta\dfrac{\pi}{\mu}}{\gamma_I + \mu} \end{pmatrix}.$$

The eigenvalues of FV^{-1} are

$$\rho_1 = 0, \quad \rho_2 = \frac{\alpha(1-f)\beta\dfrac{\pi}{\mu} + f\beta\dfrac{\pi}{\mu}(\gamma_W + \alpha + \mu)}{(\gamma_W + \alpha + \mu)(\gamma_I + \mu)}.$$

Therefore, the basic reproduction number is

$$\begin{aligned} R_0 &= \frac{\alpha(1-f)\beta\dfrac{\pi}{\mu} + f\beta\dfrac{\pi}{\mu}(\gamma_W + \alpha + \mu)}{(\gamma_W + \alpha + \mu)(\gamma_I + \mu)} \\ &= (1-f)R_0^W + fR_0^I, \end{aligned}$$

where R_0^W and R_0^I denote the reproduction numbers for the unreported and reported infected populations, respectively.

Derivation of Models with Noise

We formulate the model with additive white noise by introducing a white-noise term to each equation of the SWIRM model in (1):

$$
\begin{aligned}
dS &= (\pi - \beta(t)SI - \mu S + \phi R)dt + \sigma d\mathcal{W}(t),\\
dW &= ((1-f)\beta(t)SI - \gamma_W W - \alpha W - \mu W)dt + \sigma d\mathcal{W}(t),\\
dI &= (f\beta(t)SI + \alpha W - \gamma_I I - \mu I)dt + \sigma d\mathcal{W}(t),\\
dR &= (\gamma_W W + \gamma_I I - \mu R - \phi R)dt + \sigma d\mathcal{W}(t),\\
dM &= (\delta_S S + \delta_W W + \delta_I I + \delta_R R + \epsilon_S MS + \epsilon_W MW + \epsilon_I MI + \epsilon_R MR - \bar{\mu} M^2)dt + \sigma d\mathcal{W}(t),
\end{aligned}
\tag{5}
$$

where $\mathcal{W}(t)$ is a Wiener process and σ represents the intensity of the noise [45].

The model with multiplicative noise is described by the following stochastic differential equations:

$$
\begin{aligned}
dS &= (\pi - \beta(t)SI - \mu S + \phi R)dt + \sigma S d\mathcal{W}(t),\\
dW &= ((1-f)\beta(t)SI - \gamma_W W - \alpha W - \mu W)dt + \sigma W d\mathcal{W}(t),\\
dI &= (f\beta(t)SI + \alpha W - \gamma_I I - \mu I)dt + \sigma I d\mathcal{W}(t),\\
dR &= (\gamma_W W + \gamma_I I - \mu R - \phi R)dt + \sigma R d\mathcal{W}(t),\\
dM &= (\delta_S S + \delta_W W + \delta_I I + \delta_R R + \epsilon_S MS + \epsilon_W MW + \epsilon_I MI + \epsilon_R MR - \bar{\mu} M^2)dt + \sigma M d\mathcal{W}(t).
\end{aligned}
$$

Following the approach of Greenwood and Gordillo [47], we introduce demographic stochasticity into the SWIRM model. The population evolves in time according to the rates in Table 3.

Table 3. Transitions and rates.

Transitions	Rates
$S \to S+1$	$\pi + \phi R$
$S \to S-1$	$(1-f)\beta(t)SI + f\beta(t)SI + \mu S$
$W \to W+1$	$(1-f)\beta(t)SI$
$W \to W-1$	$(\gamma_W + \alpha + \mu)W$
$I \to I+1$	$f\beta(t)SI + \alpha W$
$I \to I-1$	$(\gamma_I + \mu)I$
$R \to R+1$	$\gamma_W W + \gamma_I I$
$R \to R-1$	$(\phi + \mu)R$
$M \to M+1$	$\delta_S S + \delta_W W + \delta_I I + \delta_R R + \epsilon_S MS + \epsilon_W MW + \epsilon_I MI + \epsilon_R MR$
$M \to M-1$	$\bar{\mu} M^2$

Each increment is expressed as the expected value of the increment plus a sum of centred increments. We illustrate this for class W:

$$
\begin{aligned}
\Delta W &= \mathbb{E}[\Delta W] + (\Delta W - \mathbb{E}[\Delta W])\\
\Delta W &= \mathbb{E}[\Delta W] + (\Delta Z_3 - \Delta Z_6 - \Delta Z_7 - \Delta Z_8),
\end{aligned}
\tag{6}
$$

where ΔZ_3, ΔZ_6, ΔZ_7 and ΔZ_8 are centred Poisson increments corresponding to infections, recovery, disease progression and natural deaths, respectively.

$$
\begin{aligned}
\mathbb{E}[\Delta W] &= \sum \Delta W P(\Delta W)\\
&= 1((1-f)\beta(t)SI)\Delta t + (-1)(\gamma_W W + \alpha W + \mu W)\Delta t\\
&= ((1-f)\beta(t)SI - \gamma_W W - \alpha W - \mu W)\Delta t
\end{aligned}
$$

Eq (6) becomes

$$
\Delta W = ((1-f)\beta(t)SI - \gamma_W W - \alpha W - \mu W)\Delta t + \Delta Z_3 - \Delta Z_6 - \Delta Z_7 - \Delta Z_8.
$$

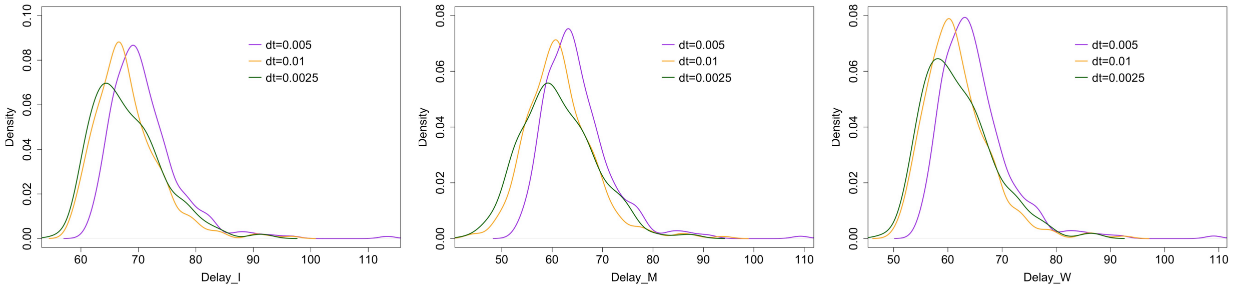

Fig 11. Density plots of delay values for variables I, M and W with the following timesteps: $dt = 0.005$ (purple), 0.01 (orange), 0.0025 (green).

We normalize the state variables so that they become proportions of the total population and replace the Poisson increments $\frac{\Delta Z_i}{N}$ by increments of Brownian motion. Then we obtain the following equations:

$$\begin{aligned}
ds =\ & (\pi/N + \phi r - (1-f)\beta(t)siN - f\beta(t)siN - \mu s)\,dt \\
& + \sqrt{\pi/N}d\mathcal{W}_1(t) + \sqrt{\phi r}d\mathcal{W}_2(t) - G_3 d\mathcal{W}_3(t) - G_4 d\mathcal{W}_4(t) - \sqrt{\mu s}d\mathcal{W}_5(t), \\
dw =\ & ((1-f)\beta(t)siN - \gamma_W w - \alpha w - \mu w)\,dt + G_3 d\mathcal{W}_3(t) \\
& - \sqrt{\gamma_W w}d\mathcal{W}_6(t) - \sqrt{\alpha w}d\mathcal{W}_7(t) - \sqrt{\mu w}d\mathcal{W}_8(t), \\
di =\ & (f\beta(t)siN + \alpha w - \gamma_I i - \mu i)\,dt + G_4 d\mathcal{W}_4(t) + \sqrt{\alpha w}d\mathcal{W}_7(t) \\
& - \sqrt{\gamma_I i}d\mathcal{W}_9(t) - \sqrt{\mu i}d\mathcal{W}_{10}(t), \\
dr =\ & (\gamma_W w + \gamma_I i - \phi r - \mu r)\,dt + \sqrt{\gamma_W w}d\mathcal{W}_6(t) + \sqrt{\gamma_I i}d\mathcal{W}_9(t) \\
& - \sqrt{\phi r}d\mathcal{W}_2(t) - \sqrt{\mu r}d\mathcal{W}_{11}(t), \\
dm =\ & (\delta_S s + \delta_W w + \delta_I i + \delta_R r + \epsilon_S msN + \epsilon_W mwN \\
& + \epsilon_I miN + \epsilon_R mrN - \bar{\mu} m^2 N)dt + G_{12} d\mathcal{W}_{12}(t) - \sqrt{\bar{\mu} m^2 N}d\mathcal{W}_{13}(t),
\end{aligned}$$

where

$$\begin{aligned}
G_3 &= \sqrt{(1-f)\beta(t)siN}, \\
G_4 &= \sqrt{f\beta(t)siN}, \\
G_{12} &= \sqrt{\delta_S s + \delta_W w + \delta_I i + \delta_R r + \epsilon_S msN + \epsilon_W mwN + \epsilon_I miN + \epsilon_R mrN}.
\end{aligned}$$

The G_i's are the standard deviations of the Poisson increments and $\mathcal{W}_i(t)$, $i = 1, 2, ..., 13$, is a Wiener process.

Sensitivity of delay values to timestep size

We conducted a sensitivity analysis to assess the effect of varying timestep size ($dt = 0.005, 0.01, 0.0025$) on the delay values for the variables I, M, and W. See Fig 11. For I, the median values remain within a narrow range ($\approx$ 67–70), while for M and W, the median values remain within a comparable range ($\approx$ 60–64), indicating limited variation in central tendency across timestep choices. In terms of dispersion, the interquartile ranges are relatively similar for $dt = 0.01$ and $dt = 0.005$, followed by an increase at $dt = 0.0025$, where the IQR expands by approximately 1.3 to 1.6 units across all variables. Overall, these quantitative differences do not affect the qualitative interpretation of the results, as delays are consistently observed across all timestep choices, indicating robustness of the underlying dynamical behaviour.
